# Deep learning classification of active tuberculosis lung zones wise manifestations using chest X-rays: a multi label approach

**DOI:** 10.1038/s41598-023-28079-0

**Published:** 2023-01-17

**Authors:** James Devasia, Hridayanand Goswami, Subitha Lakshminarayanan, Manju Rajaram, Subathra Adithan

**Affiliations:** 1 Department of Preventive and Social Medicine, Jawaharlal Institute of Postgraduate Medical Education and Research, Jipmer Campus Rd, Gorimedu, Priyadarshini Nagar, Puducherry, 605006 India; 2 Department of Pulmonary Medicine, Jawaharlal Institute of Postgraduate Medical Education and Research, Jipmer Campus Rd, Gorimedu, Priyadarshini Nagar, Puducherry, 605006 India; 3 Department of Radiodiagnosis, Jawaharlal Institute of Postgraduate Medical Education and Research, Jipmer Campus Rd, Gorimedu, Priyadarshini Nagar, Puducherry, 605006 India; 4 Department of Radiology, Marwari Hospitals, Sati Joymati Road, Athgaon, Guwahati, Assam 781008 India

**Keywords:** Tuberculosis, Computational science

## Abstract

Chest X-rays are the most economically viable diagnostic imaging test for active pulmonary tuberculosis screening despite the high sensitivity and low specificity when interpreted by clinicians or radiologists. Computer aided detection (CAD) algorithms, especially convolution based deep learning architecture, have been proposed to facilitate the automation of radiography imaging modalities. Deep learning algorithms have found success in classifying various abnormalities in lung using chest X-ray. We fine-tuned, validated and tested EfficientNetB4 architecture and utilized the transfer learning methodology for multilabel approach to detect lung zone wise and image wise manifestations of active pulmonary tuberculosis using chest X-ray. We used Area Under Receiver Operating Characteristic (AUC), sensitivity and specificity along with 95% confidence interval as model evaluation metrics. We also utilized the visualisation capabilities of convolutional neural networks (CNN), Gradient-weighted Class Activation Mapping (Grad-CAM) as post-hoc attention method to investigate the model and visualisation of Tuberculosis abnormalities and discuss them from radiological perspectives. EfficientNetB4 trained network achieved remarkable AUC, sensitivity and specificity of various pulmonary tuberculosis manifestations in intramural test set and external test set from different geographical region. The grad-CAM visualisations and their ability to localize the abnormalities can aid the clinicians at primary care settings for screening and triaging of tuberculosis where resources are constrained or overburdened.

## Introduction

Global TB report articulates 10.6 million active cases of tuberculosis (TB) [9.9–11.0, 95% uncertainty interval (UI)], 1.4 million deaths (1.3–1.5, 95% UI) and an extra 187,000 (158,000–218,000, 95% UI) deaths (TB with HIV) by a single infectious agent despite being preventable and curable^1^. Even though screening, triaging, and diagnostic methods have been developed to detect TB, early detection of Pulmonary TB continues to be challenging in clinical settings where confirmatory tests (Molecular methods, smear microscopy and culture) are limited or unavailable. Timely detection of active TB is crucial for treating an individual and for public health intervention to control TB.

Chest X-Ray (CXR) is the oldest and primary radiologic evaluation method for detecting pulmonary TB. It has high sensitivity and low specificity and may appear normal even when active TB is present^2,3^. World Health Organization mandates the use of chest X-ray in triaging and screening for active TB^4^. The integrated diagnostic algorithm in National Tuberculosis Elimination Programme in India prioritize the use of CXR as a screening method to increase the detection of active pulmonary TB followed by Nucleic Acid Amplification Test for Universal Drug Susceptible Testing. In this context, it is important for clinicians or radiologists to detect and determine the visual signals of active tuberculosis from CXR for early detection and treatment. In developing countries with high burden of TB, lack of trained clinicians in primary care settings or radiologists and inter/ intra reader variability leads to delay in diagnosing and missing out on the active Tuberculosis case^4,5^. Further, access to the existing tool and the wide systemic gaps significantly hamper reducing the risk and prevention among densely populated regions.

In this scenario, there has been an increased interest in the Artificial Intelligence community in developing Computer-Aided Diagnosis (CAD) for radiology interpretation using diverse methods which shows promising results^6–14^. Recent (2021) guidelines released by Word Health Organization recommended using CAD packages for automated screening and triage of active TB diseases among individuals above 15 years^4^ given that 56% of active TB cases developed in 2019 were individuals aged ≥ 15 years and old^1^.

The Deep Learning research community has been attracted to radiology interpretation for detecting many diseases from a single CXR. This multi label approach gained some attention due to the richness of radiological data and their coarse impressions^17^. ChestX-Ray14 dataset is widely used in the multi-label classification of various lung pathologies such as Atelectasis, Cardiomegaly, Consolidation, Edema, Effusion, Emphysema, Fibrosis, Infiltration, Mass, Nodule, Pleural Thickening, Pneumonia, and Pneumothorax^17–23^. Few other published works^6,11^ combine clinical covariant information and CXR to classify TB. Gradient-weighted class activation mapping (Grad-CAM) uses the gradients of target class abnormalities for active tuberculosis to generate a coarse heatmap highlighting important regions in chest X-ray to predict relevant manifestations in active tuberculosis. Studies have used this technique to evaluate the performance of the model^7,17,22^ but its usefulness has not been explored in this setting.

In primary care clinical settings, it is important to detect the tuberculosis abnormalities in chest X-ray that are distinguishable from other thoracic diseases for early diagnosis, treatment and management. For example, community acquired pneumonia has symptoms and radiological features similar to Tuberculosis^3,24^; this may delay in diagnosing pulmonary tuberculosis as clinicians may prescribe antibiotic for pneumonia^25,26^.

Since the inception of AlexNet, Convolutional Neural Networks (CNN) based algorithms for diagnosis of TB gained much attention compared to the classical machine learning approaches such as Random Forest or support vector machines. This CAD algorithm uses either binary^7,9,10,13,14^ (Tuberculosis vs Normal) or multi-class^15,16^ (Tuberculosis vs Normal vs other thoracic diseases) approaches to tackle tuberculosis diagnosis from chest X-ray.

In this work we aim to address these issues where we fine tune the EfficientNetB4 architecture for multi-label classification of TB specific abnormalities in lung zone wise and examine classification results through Grad-CAM visualization through radiologist perspectives. As per our best knowledge this work is one of the first attempts in the multi label classification of the manifestations of active TB with potential to widen the possibility of real-world application in primary care settings with limited resources.

## Results

### Data description

TB manifestations can be perceived in CXRs by the presence of specific patterns in the image. The abnormalities found in the CXR were organized into Parenchymal, Pleural and overall are shown in Table 1. Nearly 50.3% of the CXR exhibit pulmonary consolidation (airspace lung opacification) or cavity (thick-walled unusual gas-filled area), associated with mass or nodules primarily in bilateral upper and mid zones. Almost all CXR (99.3%) shows signs of dense patterns, ill-defined opacity or consolidation without any well-defined borders and span across all zones bilaterally. Lung manifestations of External test set (MC collection) are shown in Table 2. Opacity and fibrosis were discernable in bilateral upper and middle zone compared to lower zone. Bilateral upper zones exhibited cavitation. In MC collection right lung manifest pleural effusion and Tracheal shift than left lung.

**Table 1:** Distribution of radiological abnormalities—lung wise and zone wise representation used in the model.

Abnormality in CXR	Lung wise	Right lung			Left lung		
		RUZ	RMZ	RLZ	LUZ	LMZ	LLZ
Parenchymal							
Opacity	1303 (99.3)	1003 (76.5)	986 (75.2)	765 (58.3)	888 (67.7)	949 (72.3)	777 (59.2)
Fibrosis	725 (55.3)	513 (39.1)	331 (25.2)	133 (10.1)	452 (34.5)	389 (29.7)	142 (10.8)
Collapsed lung	710 (54.1)	423 (32.2)	277 (21.1)	246 (18.8)	299 (22.8)	239 (18.2)	222 (16.9)
Cavity	660 (50.3)	361 (27.5)	215 (16.4)	38 (2.9)	274 (20.9)	219 (16.7)	36 (2.7)
Calcification	142 (10.8)	105 (8.0)	104 (7.9)	35 (2.8)	82 (6.3)	93 (7.1)	22 (1.7)
Nodule*	81 (6.2)	38 (3.0)	33 (2.5)	22 (1.7)	40 (3.1)	39 (3.0)	20 (1.5)
Hilar adenopathy*	74 (5.6)	55 (4.2)	56 (4.3)	55 (4.2)	23 (1.6)	23 (1.8)	23 (1.8)
Interstitial thickening*	32 (2.4)	27 (2.1)	29 (2.2)	28 (2.1)	28 (2.1)	31 (2.7)	29 (2.2)
Bronchiectasis*	16 (1.2)	10 (0.8)	4 (0.3)	0 (0)	5 (0.4)	2 (0.2)	0 (0)
Pleural							
Thickening	688 (52.4)		426 (32.5)			411 (31.3)	
Pneumo/hydro pneumothorax	205 (15.6)		102 (7.8)			104 (7.9)	
Effusion/loculated collection	180 (13.7)		121 (9.2)			80 (6.1)	
Overall							
Tracheal shift	537 (40.9)		376 (28.7)			161 (12.3)	
Volume loss	461 (35.1)		276 (21.0)			219 (16.7)	
Mediastinal shift	200 (15.2)		106 (8.1)			94 (7.2)	
Emphysema/hyperinflations	132 (10.1)		123 (9.4)			124 (9.5)	
Mediastinal widening*	60 (4.6)		35 (2.7)			31 (2.4)	
Surgical emphysema*	53 (4.0)		27 (2.1)			32 (2.4)	
Hilar shift*	22 (1.7)		10 (0.8)			12 (0.9)	

*RUZ* right upper zone, *RMZ* right mid zone, *RLZ* right lower zone, *LUZ* left upper zone, *LMZ* left mid zone, *LLZ* left lower zone.

*< 10% distribution and hence excluded from model development. All values represented n (%) format.

**Table 2:** Distribution of radiological abnormalities in external test set—lung wise and zone wise representation used in the model.

Abnormality in CXR	Right lung			Left lung		
	RUZ	RMZ	RLZ	LUZ	LMZ	LLZ
Parenchymal						
Opacity	12 (33.3)	8 (22.2)	5 (13.8)	9 (25.0)	6 (16.7)	1 (2.8)
Cavity	9 (25.0)	0 (0.0)	0 (0.0)	4 (11.1)	3 (8.3)	0 (0.0)
Fibrosis	7 (19.4)	2 (5.6)	0 (0.0)	5 (13.8)	2 (5.6)	0 (0.0)
Calcification	0 (0.0)	0 (0.0)	1 (2.8)	2 (5.6)	1 (2.8)	0 (0.0)
Collapsed lung	0 (0.0)	1 (2.8)	0(0.0)	1 (2.8)	0 (0.0)	0 (0.0)
Pleural						
Effusion/loculated collection		8 (22.2)			3 (8.3)	
Thickening		2 (5.6)			2 (5.6)	
Pneumo/hydro pneumothorax		0 (0.0)			0 (0.0)	
Overall						
Tracheal shift		4 (11.1)			0 (0.0)	
Volume loss		1 (2.8)			1 (2.8)	
Mediastinal shift		1 (2.8)			0 (0.0)	
Emphysema/hyperinflations		0 (0.0)			0 (0.0)	

All values represented n (%) format.

*RUZ* right upper zone, *RMZ* right mid zone, *RLZ* right lower zone, *LUZ* left upper zone, *LMZ* left mid zone, *LLZ* left lower zone.

### Results from image wise abnormality

**Figure 1:**
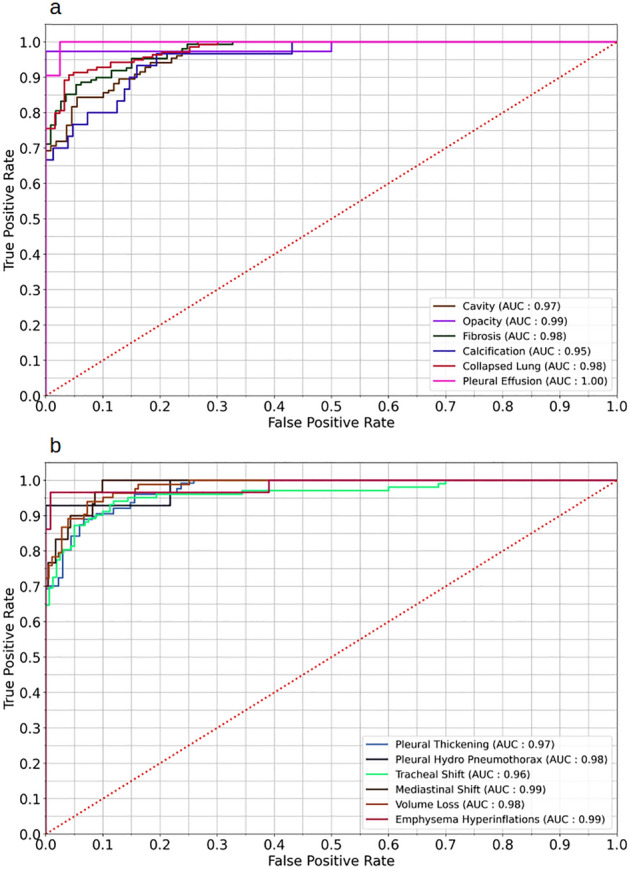
Receiver operating characteristic of lung wise abnormalities. (**a**) Area under receiver operating characteristic (AUC) of cavity, opacity, fibrosis, calcification, collapsed lung, pleural effusion. (**b**) AUC of pleural thickening, pneumo/hydropneumothorax, tracheal shift, mediastinal shift, volume loss, emphysema hyperinflations.

The Area Under the Receiver Operating Characteristic (AUC), sensitivity, and specificity of the model for holdout test set for image wise TB abnormalities are shown in Table 3. AUC ranges from 0.95 [95% CI: 0.90–1.00] to 1.00 [95% CI: 0.98–1.00] with Calcification showing lowest AUC of 0.95 [95% CI 0.90–1.00] and Pleural Effusion with a perfect AUC of 1.00 [95% CI 0.98–1.00]. Figure 1 shows the Receiver Operating Characteristic (ROC) curve for image wise abnormalities. The sensitivity and specificity of image wise abnormality varies from 0.70 to 1.00 and 0.50–1.00 respectively. Calcification and Mediastinal Shift shows the least sensitivity of 0.70 [95% CI 0.54–0.86] and in specificity, except Opacity 0.50 [95% CI 0.00–1.00] other 11 abnormalities reported specificity above 0.90. The number of True Negatives (n = 2) elucidates the reason for low specificity in Opacity. Supplementary Table S1 shows the accuracy, precision and F1 Score of 12 image wise manifestations, accuracy ranges from 87 to 100% with Opacity and Pneumothorax /Hydropneumothorax scores of 100% accuracy. Precision, the ability to detect a sample as positive changes from 81 to 100% with Opacity, Pneumothorax /Hydropneumothorax and Emphysema Hyperinflations scored 100%. F1 Score for the model diverges between 0.81 to 1.00 and Opacity has F1 score of 1.00.

**Table 3:** Estimates of diagnostic metrics of lung wise abnormalities in EfficientNetB4 model.

Abnormality in CXR	AUC [95% CI]	Sensitivity [95% CI]	Specificity [95% CI]
Cavity	0.97 [0.94–0.99]	0.82 [0.76–0.88]	0.94 [0.90–0.99]
Opacity	0.99 [0.97–1.00]	1.00 [1.00–1.00]	0.50 [0.00–1.00]
Fibrosis	0.98 [0.96–0.99]	0.89 [0.84–0.94]	0.92 [0.87–0.97]
Calcification	0.95 [0.90–1.00]	0.70 [0.54–0.86]	0.98 [0.96–1.00]
Collapsed lung	0.98 [0.96–1.00]	0.87 [0.81–0.93]	0.96 [0.93–0.99]
Pleural effusion	1.00 [0.98–1.00]	0.90 [0.78–1.00]	1.00 [0.99–1.00]
Pleural thickening	0.97 [0.95–0.99]	0.89 [0.84–0.94]	0.93 [0.89–0.98]
Pneumo/hydropneumothorax	0.98 [0.94–1.00]	0.93 [0.79–1.00]	1.00 [1.00–1.00]
Tracheal shift	0.96 [0.93–0.99]	0.81 [0.74–0.89]	0.96 [0.92–0.99]
Mediastinal shift	0.99 [0.96–1.00]	0.70 [0.54–0.86]	1.00 [0.99–1.00]
Volume loss	0.98 [0.96–1.00]	0.87 [0.79–0.94]	0.97 [0.94–0.99]
Emphysema hyperinflations	0.99 [0.96–1.00]	0.86 [0.74–0.99]	1.00 [1.00–1.00]

95% CI for AUC calculated using Hanley & McNeil method. 95% CI for sensitivity and specificity calculated using Wilson Score method.

*AUC* area under the receiver operating characteristic, *CI* confidence interval.

### Results from lung wise and lung zone wise abnormalities

Table 4 depicts the AUC, sensitivity and specificity of lung zone wise (upper, middle and lower) and lung wise (right and left) abnormalities. Out of 44 abnormalities, 52.2% (23) manifestation has AUC > 0.90 and 21 manifestations have AUC between 0.76 and 0.89. Cavity Left Lower Zone has the lowest AUC of 0.56 [95% CI 0.33–0.78]. Bilateral lower zone Cavity and Calcification left lower zone revealed zero sensitivity mainly because of the lower number of data points i.e. 2.90% (n = 38), 2.74% (n = 36) and 1.68% (n = 22) respectively. We spotted that 52.2% (n = 23) manifestations has sensitivity above 70% and 41% (n = 18) has sensitivity varying from 18.00 to 69.00%. Specificity ranges from 60 to 100% with 80% (n = 35) manifestations having specificity above 90% and rest of the abnormalities has specificity varies from 60 to 89%. The external test set (n = 36) validation (Table 5) shows an AUC ranging from 0.31 [0.00–0.64] to 1.00 [1.00–1.00] for (n = 25) various abnormalities. Nineteen abnormalities exhibited zero true positive and excluded from the analysis. Out of 25 abnormalities, 88% (n = 22) scored AUC > 0.80. Nearly 50% of the abnormality scored perfect sensitivity with 100%. Specificity for the external test set ranges from 0.58 [0.41–0.75] to 1.00 [1.00–1.00] for 44 abnormalities −80% (n = 36) scored specificity > 80% with nearly 50% (n = 12) scored perfect score of 100%.

**Table 4:** Estimates of diagnostic metrics of lung zone wise abnormalities in EfficientNetB4 model.

Abnormality in CXR	AUC [95% CI]	Sensitivity [95% CI]	Specificity [95% CI]
Cavity RUZ	0.89 [0.84–0.93]	0.68 [0.58–0.77]	0.88 [0.83–0.93]
Cavity LUZ	0.89 [0.84–0.94]	0.70 [0.59–0.80]	0.87 [0.82–0.92]
Cavity RMZ	0.87 [0.80–0.94]	0.54 [0.40–0.69]	0.91 [0.87–0.95]
Cavity LMZ	0.81 [0.73–0.89]	0.44 [0.30–0.58]	0.93 [0.89–0.96]
Cavity RLZ	0.82 [0.67–0.97]	0.00 [0.00–0.00]	0.99 [0.98–1.00]
Cavity LLZ	0.56 [0.33–0.78]	0.00 [0.00–0.00]	0.99 [0.98–1.00]
Opacity RUZ	0.84 [0.79–0.89]	0.83 [0.78–0.88]	0.66 [0.53–0.79]
Opacity LUZ	0.89 [0.86–0.93]	0.78 [0.73–0.84]	0.90 [0.83–0.97]
Opacity RMZ	0.80 [0.74–0.85]	0.79 [0.74–0.85]	0.60 [0.49–0.72]
Opacity LMZ	0.88 [0.84–0.92]	0.85 [0.80–0.90]	0.78 [0.68–0.88]
Opacity RLZ	0.80 [0.75–0.86]	0.66 [0.59–0.74]	0.84 [0.77–0.90]
Opacity LLZ	0.87 [0.83–0.92]	0.71 [0.63–0.78]	0.88 [0.81–0.94]
Fibrosis RUZ	0.93 [0.89–0.96]	0.81 [0.74–0.88]	0.91 [0.86–0.95]
Fibrosis LUZ	0.88 [0.84–0.93]	0.73 [0.64–0.82]	0.90 [0.85–0.94]
Fibrosis RMZ	0.81 [0.74–0.88]	0.49 [0.37–0.62]	0.88 [0.83–0.92]
Fibrosis LMZ	0.78 [0.72–0.85]	0.60 [0.49–0.71]	0.75 [0.69–0.81]
Fibrosis RLZ	0.76 [0.65–0.87]	0.23 [0.07–0.39]	0.95 [0.92–0.98]
Fibrosis LLZ	0.77 [0.68–0.87]	0.23 [0.09–0.37]	0.98 [0.97–1.00]
Calcification RUZ	0.93 [0.86–1.00]	0.65 [0.46–0.85]	0.97 [0.96–0.99]
Calcification LUZ	0.92 [0.83–1.00]	0.53 [0.29–0.77]	0.97 [0.95–0.99]
Calcification RMZ	0.92 [0.83–1.00]	0.71 [0.52–0.91]	0.98 [0.97–1.00]
Calcification LMZ	0.92 [0.83–1.00]	0.47 [0.25–0.70]	0.98 [0.96–0.99]
Calcification RLZ	0.84 [0.69–0.98]	0.18 [0.00–0.41]	1.00 [1.00–1.00]
Calcification LLZ	0.78 [0.56–1.00]	0.00 [0.00–0.00]	1.00 [0.99–1.00]
Collapsed lung RUZ	0.92 [0.88–0.96]	0.71 [0.61–0.81]	0.91 [0.86–0.95]
Collapsed lung LUZ	0.95 [0.91–0.99]	0.74 [0.63–0.85]	0.94 [0.91–0.98]
Collapsed lung RMZ	0.83 [0.74–0.92]	0.36 [0.20–0.53]	0.93 [0.90–0.96]
Collapsed lung LMZ	0.92 [0.86–0.98]	0.69 [0.54–0.84]	0.93 [0.90–0.97]
Collapsed lung RLZ	0.90 [0.82–0.99]	0.45 [0.25–0.66]	0.96 [0.94–0.99]
Collapsed lung LLZ	0.91 [0.84–0.98]	0.55 [0.38–0.72]	0.97 [0.94–0.99]
Pleural effusion R	0.98 [0.92–1.00]	1.00 [1.00–1.00]	0.95 [0.92–0.98]
Pleural effusion L	0.99 [0.95–1.00]	0.92 [0.76–1.00]	0.98 [0.97–1.00]
Pleural thickening R	0.91 [0.87–0.96]	0.69 [0.59–0.79]	0.91 [0.87–0.95]
Pleural thickening L	0.94 [0.90–0.98]	0.85 [0.77–0.93]	0.90 [0.86–0.94]
Pneumo/hydropneumothorax R	1.00 [0.96–1.00]	0.75 [0.45–1.00]	1.00 [0.99–1.00]
Pneumo/hydropneumothorax L	0.89 [0.72–1.00]	0.83 [0.54–1.00]	0.99 [0.98–1.00]
Tracheal shift R	0.93 [0.89–0.97]	0.78 [0.68–0.88]	0.92 [0.88–0.96]
Tracheal shift L	0.94 [0.89–1.00]	0.76 [0.62–0.91]	0.97 [0.95–0.99]
Mediastinal shift R	0.96 [0.88–1.00]	0.62 [0.35–0.88]	0.99 [0.97–1.00]
Mediastinal shift L	0.98 [0.93–1.00]	0.71 [0.49–0.92]	0.98 [0.96–1.00]
Volume loss R	0.97 [0.93–1.00]	0.76 [0.64–0.88]	0.96 [0.94–0.99]
Volume loss L	0.98 [0.95–1.00]	0.90 [0.80–0.99]	0.98 [0.96–1.00]
Emphysema R	0.96 [0.91–1.00]	0.88 [0.75–1.00]	0.97 [0.95–0.99]
Emphysema L	0.99 [0.96–1.00]	0.92 [0.81–1.00]	0.97 [0.95–0.99]

95% CI for AUC calculated using Hanley & McNeil method. 95% CI for sensitivity and specificity calculated using Wilson Score method.

*AUC* area under the receiver operating characteristic, *RUZ* right upper zone, *RMZ* right mid zone, *RLZ* right lower zone, *LUZ* left upper zone, *LMZ* left mid zone, *LLZ* left lower zone, *R* right lung, *L* left lung, *CI* confidence interval.

**Table 5:** Estimates of diagnostic metrics of lung zone wise abnormalities in EfficientNetB4 model in external test set.

Abnormality	AUC [95% CI]	Sensitivity [95% CI]	Specificity [95% CI]
Cavity RUZ	0.98 [0.92–1.00]	0.44 [0.12–0.77]	1.00 [1.00–1.00]
Cavity LUZ	0.86 [0.62–1.00]	0.25 [0.00–0.67]	0.97 [0.91–1.00]
Cavity RMZ	–	–	0.97 [0.92–1.00]
Cavity LMZ	0.89 [0.64–1.00]	0.00 [0.00–0.00]	1.00 [1.00–1.00]
Cavity RLZ	–	–	1.00 [1.00–1.00]
Cavity LLZ	–	–	1.00 [1.00–1.00]
Opacity RUZ	0.81 [0.65–0.97]	0.83 [0.62–1.00]	0.63 [0.43–0.82]
Opacity LUZ	0.89 [0.74–1.00]	0.56 [0.23–0.88]	0.96 [0.89–1.00]
Opacity RMZ	0.91 [0.77–1.00]	1.00 [1.00–1.00]	0.64 [0.47–0.82]
Opacity LMZ	0.98 [0.91–1.00]	1.00 [1.00–1.00]	0.93 [0.84–1.00]
Opacity RLZ	0.88 [0.69–1.00]	1.00 [1.00–1.00]	0.58 [0.41–0.75]
Opacity LLZ	0.94 [0.62–1.00]	1.00 [1.00–1.00]	0.94 [0.87–1.00]
Fibrosis RUZ	0.85 [0.67–1.00]	0.57 [0.20–0.94]	0.86 [0.74–0.99]
Fibrosis LUZ	0.89 [0.70–1.00]	0.80 [0.45–1.00]	0.81 [0.67–0.95]
Fibrosis RMZ	0.94 [0.71–1.00]	1.00 [1.00–1.00]	0.88 [0.77–0.99]
Fibrosis LMZ	0.88 [0.57–1.00]	1.00 [1.00–1.00]	0.74 [0.59–0.88]
Fibrosis RLZ	–	–	0.94 [0.87–1.00]
Fibrosis LLZ	–	–	1.00 [1.00–1.00]
Calcification RUZ	–	–	1.00 [1.00–1.00]
Calcification LUZ	0.31 [0.00–0.64]	0.00 [0.00–0.00]	1.00 [1.00–1.00]
Calcification RMZ	–	–	1.00 [1.00–1.00]
Calcification LMZ	0.49 [0.00–1.00]	0.00 [0.00–0.00]	1.00 [1.00–1.00]
Calcification RLZ	–	0.00 [0.00–0.00]	1.00 [1.00–1.00]
Calcification LLZ	–	–	1.00 [1.00–1.00]
Collapsed lung RUZ	–	–	0.92 [0.83–1.00]
Collapsed lung LUZ	1.00 [1.00–1.00]	1.00 [1.00–1.00]	0.97 [0.92–1.00]
Collapsed lung RMZ	0.97 [0.74–1.00]	1.00 [1.00–1.00]	0.94 [0.87–1.00]
Collapsed lung LMZ	–]	–	1.00 [1.00–1.00]
Collapsed lung RLZ	–	–	0.97 [0.92–1.00]
Collapsed lung LLZ	–	–	1.00 [1.00–1.00]
Pleural effusion R	1.00 [1.00–1.00]	1.00 [1.00–1.00]	1.00 [1.00–1.00]
Pleural effusion L	0.84 [0.55–1.00]	0.67 [0.13–1.00]	1.00 [1.00–1.00]
Pleural thickening R	0.68 [0.25–1.00]	0.50 [0.00–1.00]	0.91 [0.82–1.00]
Pleural thickening L	0.97 [0.80–1.00]	1.00 [1.00–1.00]	0.97 [0.91–1.00]
Pneumo/hydropneumothorax R	–	–	1.00 [1.00–1.00]
Pneumo/hydropneumothorax L	–	–	1.00 [1.00–1.00]
Tracheal shift R	0.97 [0.85–1.00]	0.75 [0.33–1.00]	0.97 [0.91–1.00]
Tracheal shift L	–	–	1.00 [1.00–1.00]
Mediastinal shift R	1.00 [1.00–1.00]	1.00 [1.00–1.00]	1.00 [1.00–1.00]
Mediastinal shift L	–	–	1.00 [1.00–1.00]
Volume loss R	1.00 [1.00–1.00]	0.00 [0.00–0.00]	1.00 [1.00–1.00]
Volume loss L	0.97 [0.74–1.00]	1.00 [1.00–1.00]	0.97 [0.92–1.00]
Emphysema R	–	–	0.94 [0.87–1.00]
Emphysema L	–	–	0.89 [0.79–0.99]

95% CI for AUC calculated using Hanley & McNeil method. 95% CI for sensitivity and specificity calculated using Wilson Score method. – indicate no abnormalities found.

*AUC* area under the receiver operating characteristic, *RUZ* right upper zone, *RMZ* right mid zone, *RLZ* right lower zone, *LUZ* left upper zone, *LMZ* left mid zone, *LLZ* left lower zone, *R* right lung, *L* left lung, *CI* confidence interval.

**Figure 2:**
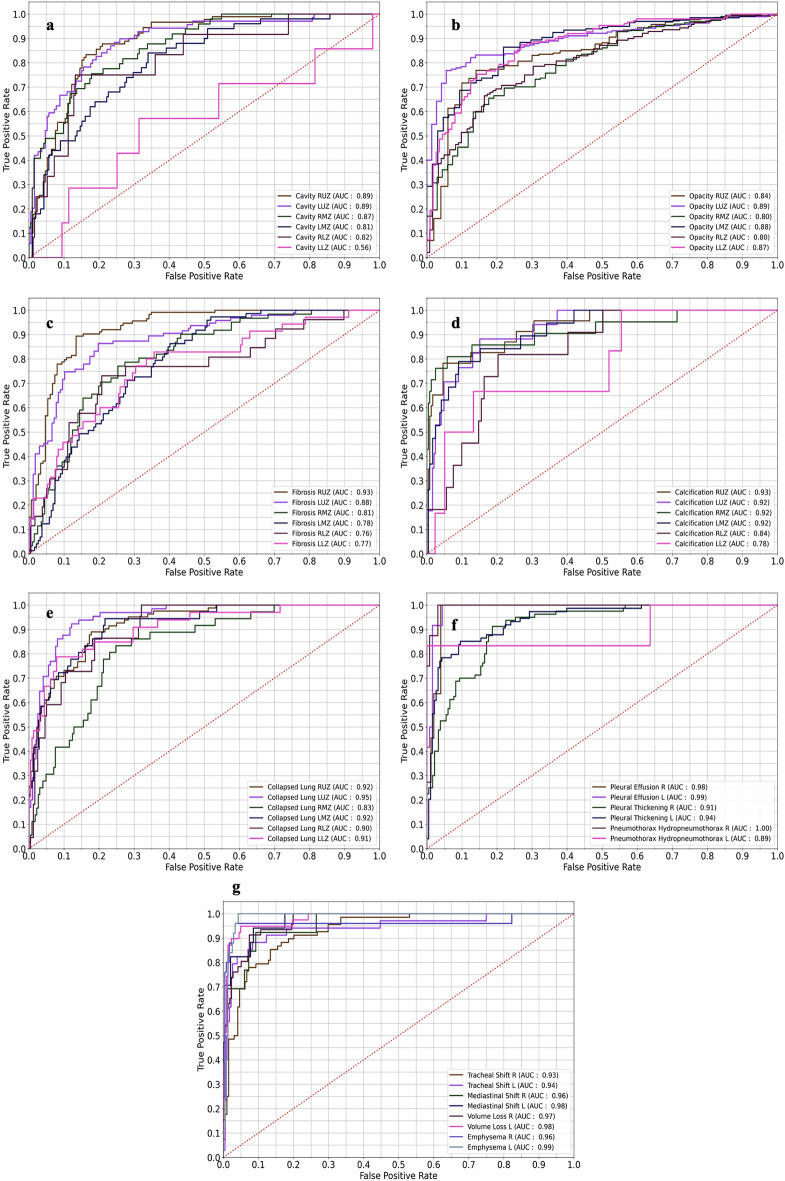
Receiver operating characteristic of lung zone wise abnormalities. (**a**) Area under receiver operating characteristic (AUC) of cavity. (**b**) AUC of opacity. (**c**) AUC of fibrosis. (**d**) AUC of calcification. (**e**) AUC of collapsed lung. (**f**) AUC of pleural effusion, pleural thickening, pneumo/hydropneumothorax. (**g**) AUC of tracheal shift, mediastinal shift, volume loss, emphysema. *RUZ* right upper zone, *RMZ* right mid zone, *RLZ* right lower zone, *LUZ* left upper zone, *LMZ* left mid zone, *LLZ* left lower zone, *R* right lung, *L* left lung.

**Figure 3:**
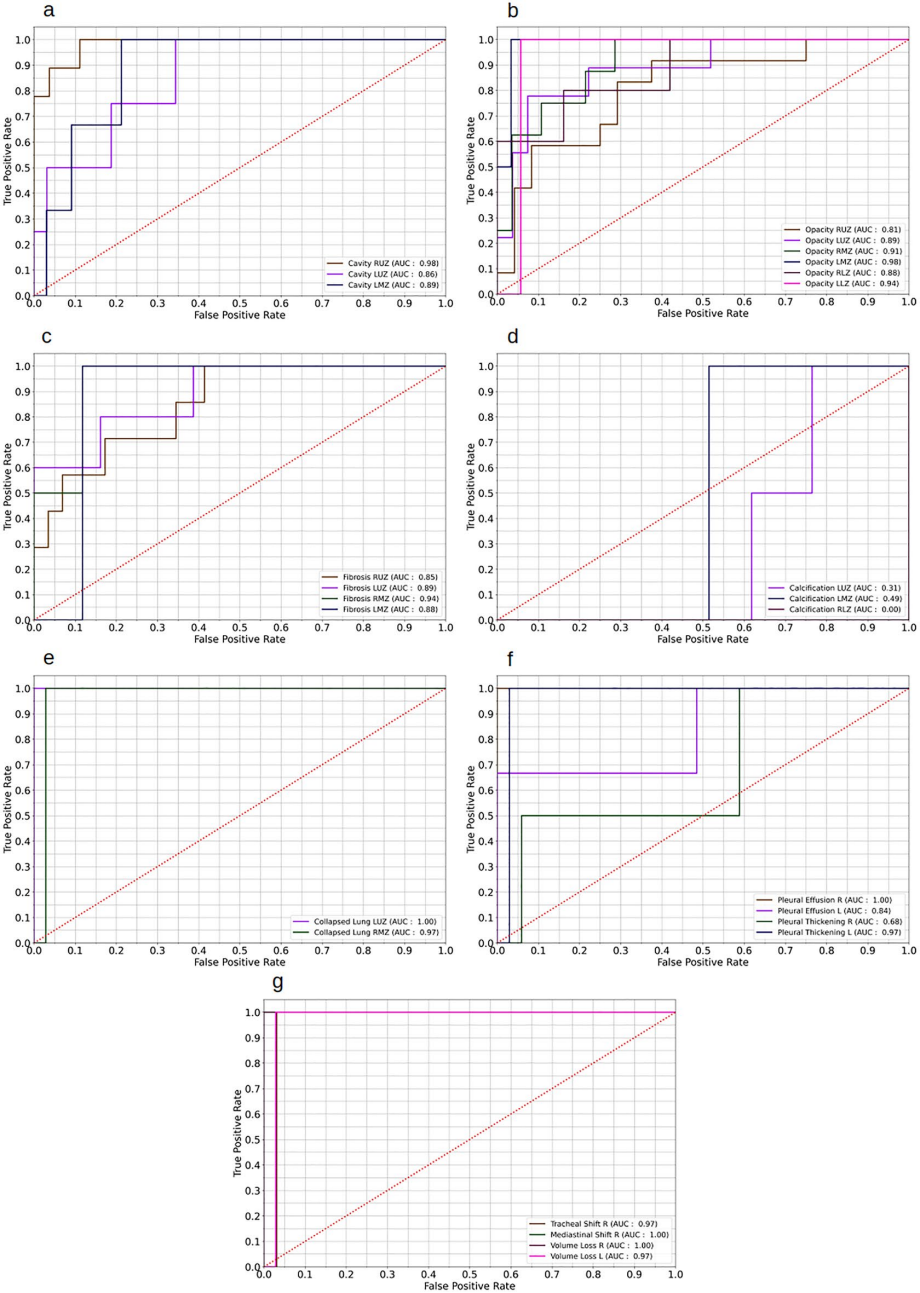
Receiver operating characteristic of lung zone wise abnormalities in external test set. (**a**) Area under receiver operating characteristic (AUC) of cavity. (**b**) AUC of opacity. (**c**) AUC of fibrosis. (**d**) AUC of calcification. (**e**) AUC of collapsed lung. (**f**) AUC of pleural effusion, pleural thickening. (**g**) AUC of tracheal shift, mediastinal shift, volume loss. *RUZ* right upper zone, *RMZ* right mid zone, *RLZ* right lower zone, *LUZ* left upper zone, *LMZ* left mid zone, *LLZ* left lower zone, *R* right lung, *L* left lung.

Supplementary Table S2 shows the accuracy, precision and F1 score of 44 manifestations of intramural holdout test set. Accuracy varies between 71 and 99% with 50% (n = 22) abnormalities having accuracy above 90%. F1 score for bilateral lower zone Cavity and Calcification left lower zone were declared as undefined. Accuracy, precision and F1 score for external test set (supplementary Table S3) shows that accuracy ranges from 64 to 100%, 72% abnormalities scored accuracy > 90% with nearly 30% (n = 14) abnormalities spotted with 100% accuracy. Due to the lack of true positive data points, many abnormalities were declared undefined in precision and F1 score. ROC curve for lung zone wise and lung wise abnormalities for intramural test set (Fig. 2) and external test set are also shown(Fig. 3).

### Grad-CAM analysis

In this section we examine the performance of grad-CAM image to visually analyse the pathological changes in the CXR (ground truth) thereby visualizing the signals of Tuberculosis. Grad-CAMs can be generated in any layer in the network, and offer better localization than saliency maps when taken in deeper layers^27^. The samples presented in this section have been generated with the network trained on the dataset with 44 classes. Supplementary Fig. S2 provides the grad-CAM images of active tuberculosis manifestations generated with network trained on the dataset with lung zone wise and lung wise manifestations, to prove the usefulness of heatmap images and coarse localisation.

### Grad-CAM analysis of intramural holdout test set

**Figure 4:**
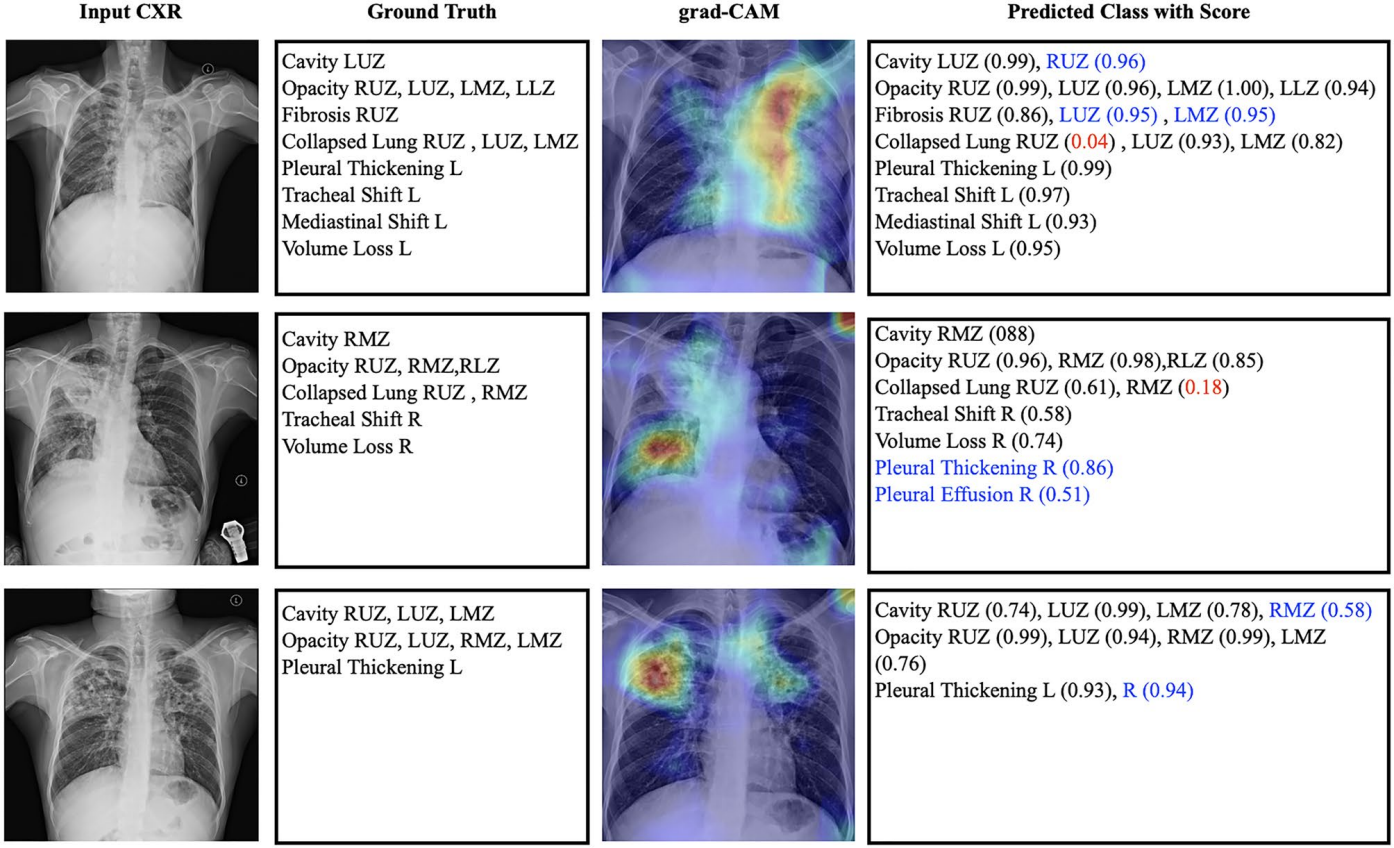
Grad-CAM image overlaid with chest Xray of Tuberculosis patients. Panel (Input CXR) shows the chest radiographic images of patients. Panel (ground truth) shows the radiologist findings of abnormalities in CXR. Panel (grad-CAM) shows the gradient-weighted class activation map overlaid with input CXR image. Panel (predicted class with score) shows the abnormality prediction with class score 0 to 1. Red coloured class prediction is false negative. Blue coloured prediction is additional findings (either true positive or false positive). *RUZ* right upper zone, *RMZ* right mid zone, *RLZ* right lower zone, *LUZ* left upper zone, *LMZ* left mid zone, *LLZ* left lower zone, *R* right lung, *L* left lung.

Figure 4 shows 45-year-old male with confirmed active pulmonary tuberculosis. The CXR shows cavity lesion in left upper zone along with patchy opacities. Opacity is found in left lung all zones and right upper zone. Fibrotic scars are seen in right upper zone with appearance of lung collapse. CXR also reveals the collapsed lung in left upper and mid zones, and the left lung appears to have volume loss and pleural thickening. Due to the abnormal pressure in the chest cavity on left upper zone, Tracheal shift towards to left is also visible in CXR. CXR also shows signs of severe build-up asymmetry of intrathoracic pressures and mediastinum (the heart, trachea, great vessels and esophagus to shift towards left). The network predicted class with scores shows that all manifestations are correctly predicted and are aligned with radiologist (HG) findings, except collapsed lung in right upper zone (score 0.04 with maximum 1). In addition, thenetwork also predicted Cavity lesion in right upper zone, Fibrotic scars in upper and mid zones of left lung. The grad-CAM heat maps are in line with ground truth focused on left lung and right mediastinal borders. Heatmap also shows the signs in right lower zone and in cardiomediastinal contour areas which is over estimated.

The second row of Fig. 4 represents X ray of 37-year-old male X-ray withGround truth showing lung abnormalities in right hemithorax. Chest X-ray showed the ill-defined patchy opacity in right lung with Cavitation in right mid zone. Right upper and mid zone exhibited lung collapse and shows signs of volume loss in right lung. Tracheal deviation towards the right side also visible due to the abnormal pressure on chest. The algorithm predictions are in line with ground truth except the false negative on Collapsed lung on right mid zone (score 0.18). Network also predicted Pleural thickening on right with score of 0.86 and Pleural Effusion on right lung (score 0.51) are verified and confirmed with radiologist (HG). The grad-CAM heatmap analysis also points to the ground truth abnormalities on left diaphragm and the left mediastinal border of left hemithorax. The coarse localisation of the predicted abnormalities can be visualized in heatmap. The Opacity on right lower Zone was highlighted and overall the heatmap wasin accordance with ground truth and verified by radiologist.

The sample in third row in Fig. 4 shows 30-year-old female patient confirmed with active pulmonary tuberculosis. The ground truth shows that the pathological changes in X-ray are bilateral upper zone and left mid zone Cavitation, Opacity in bilateral upper and mid zones and Pleural Thickening on left lung. Network predicted all classes to be consistent with ground truth and in addition Cavity lesion on right mid zone (score 0.58) and right lung Pleural Thickening (score 0.94) which is verified and agreed by radiologist. Heatmap produced by the algorithm was analysed by radiologist and showed alignment with ground truth. Some over estimation on right lower zone in grad-CAM heatmap was noticed.

### Grad-CAM analysis of external test set

**Figure 5:**
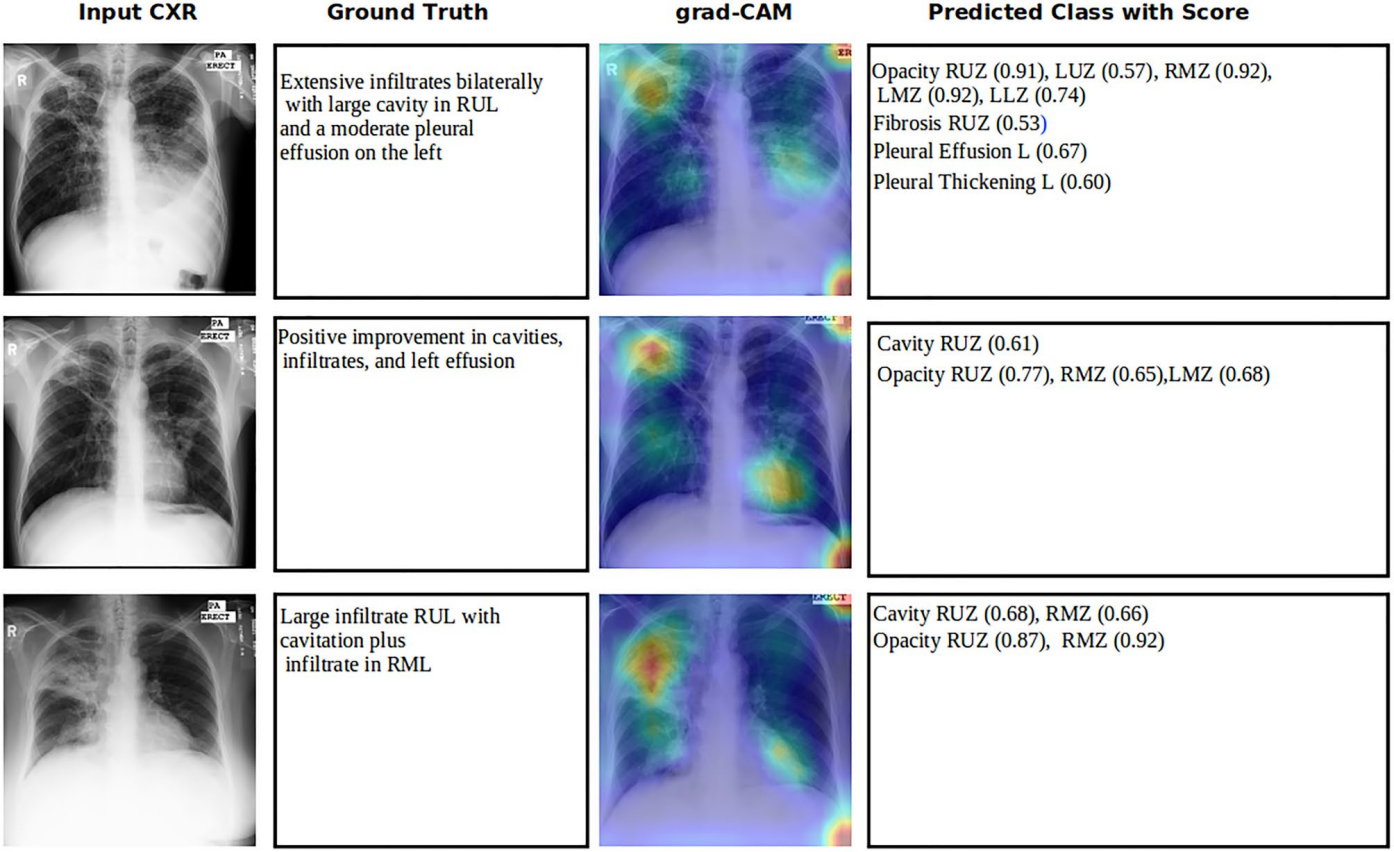
Grad-CAM image overlaid with Tuberculosis patients in external test set. Panel (input CXR) shows the chest X-ray images of patients. Panel (ground truth) shows the clinical readings associated with CXR. Panel (grad-CAM) shows the gradient-weighted class activation map overlaid with input CXR image. Panel (predicted class with score) shows the abnormality prediction with class score 0 to 1. *RUZ* right upper zone, *RMZ* right mid zone, *LUZ* left upper zone, *LMZ* left mid zone, *LLZ* left lower zone, *L* left lung.

The sample image in the first row in Fig. 5 shows 32-year-old male patient confirmed Acid-Fast Bacilli (AFB) smear and NAAT testing positive for *Mycobacterium tuberculosis* (MTB). This CXR shows bilateral infiltrates with cavity in right upper lobe and pleural effusion on left lung. Network predicted extensive infiltration except right lower zone and missed the cavity in RUL. Pleural effusion predicted with score of 0.67 and in addition, right upper zone fibrotic scars (0.53) and pleural thickening on left lung (0.60) were also predicted. Heat map analysis revealed cavity in RUL, bilateral infiltration which are aligned with ground truth. Right lung blunted costophrenic angle showed up in heatmap which was over estimated as confirmed with radiologist.

Second row in Fig. 5 is repeated (unknown duration) image of the same patient in first row sample with ground truth uncovering pathological improvements in cavities, infiltrations and left effusion. Network prediction aligned with clinical findings. Heatmap analysis reviewed by radiologist expressed cavity in RUZ prediction aligned with heatmap and coarse estimation of opacity in RUZ, RMZ and LMZ. Heat generated near left ventricle and left atrium are declared as noise.

Figure 5 third row sample shows chest X-ray of 63 years old female with active cavitary TB. Findings show large infiltration with cavitation in right upper lobe and signs of infiltration in right middle lobe. The algorithm prediction aligned with ground truth with cavity in RUZ and RMZ (scores of 0.68 and 0.66 respectively) and opacity in RUZ and RMZ (scores of 0.87 and 0.92 respectively). Grad cam heatmap verified by radiologist stressed that heat spread to left lung is noise and heatmap pointed to the cavitational area. It is also noted that the spread of the heat from RMZ to RLZ is turned to an over estimation of abnormality.

## Discussion

Tuberculosis in adults’ manifests as assorted findings in chest X rays, often cavitation and ill-defined opacity in the apical zone and broncho-pulmonary segments of upper and lower lobes. Cavitation is the hallmark in adult active TB and exhibits in half of the cases^2,3,28,29^. In this study, the EfficientNetB4 architecture for multi-label classification of TB specific abnormalities and Grad-CAM visualization has shown a good performance in localising the varied manifestations of TB.

In an accuracy study conducted in a tertiary hospital in India^30^, deep learning algorithm (qXR, Qure.ai, Mumbai, India) shows the AUC for Pleural Effusion as 0.94 (95% CI: 0.92–0.96) and for Cavity as 0.84 (95% CI: 0.82–0.87). Our work outperformed qXR in detecting Pleural Effusion and Cavity with AUC of 1.00 [95% CI 0.98–1.00] and 0.97 [95% CI: 0.94–0.99] respectively. The other comparable abnormalities were detection of Opacity and Fibrosis, qXR achieved AUC 0.88 and 0.86 while our work outperformed qXR in AUC with 0.99 and 0.98 respectively. Another feasibility study^31^ using commercial-grade deep learning software ViDi (Suite v2.0; ViDi Systems, Villaz-Saint-Pierre, Switzerland), reported AUC for Cavity, Pleural Effusion and Consolidation (mainly Opacity without defined border) as 0.90, 1.00 and 0.75 in their validation set. Our work shows better AUC for Cavity and Consolidation and comparable AUC for Pleural Effusion. Our work also achieved superior sensitivity, specificity and precision compared to ViDi for Cavity—our network achieved 82% vs. 50% for sensitivity, 94% vs. 92% for specificity and 95% vs 25% for precision. Similarly, for Opacity our model showed 100% vs 74% for sensitivity, 50% vs 76% in specificity and 100% vs 74% for precision. In the case of Pleural Effusion, both the models are comparable with specificity (both networks at 100%), while lower sensitivity (90% EfficientNetB4 vs 100% ViDi) and precision (95% EfficientNetB4 vs 100% ViDi).

On comparison with another Ensembled solution of six pre-trained networks (VGG16, VGG19, Inception V3, ResNet34, ResNet50, and ResNet101)^32^, our work shows superior performance in terms of overall AUC and accuracy. Comparable performance on AUC was noted for Opacity/Consolidation (0.99 vs 0.97 for EfficientNetB4 vs ensemble model), Pleural Effusion (1.00 vs 0.99), Fibrosis (0.98 vs 0.97) and Pleural Thickening was (0.97 vs 0.98). Superior accuracy was noted for Opacity/Consolidation 100% vs 84%, Pleural Effusion 99% vs 89%, Fibrosis 90% vs 87% and Pleural Thickening 91% vs 86%. Our work achieved superior sensitivity, specificity and precision compared to the sensitivity, specificity and precision of validation set in the feasibility study^31^. For Cavity our network achieved sensitivity of 82% while ViDi scored 50% for sensitivity, for specificity ours was 94% and ViDi was 92% and for precision ours was 95% and ViDi able to obtain 25% precision. Similarly, Opacity 100% vs 74% for sensitivity, 50% vs 76% in specificity and for precision 100% vs 74%. In case of Pleural Effusion, the results on are par with our findings, sensitivity 90% vs 100%, specificity match with both networks 100% and precision was 95% vs 100%^31^.

The external test set for validation is taken from different geographical region than the training, validation and test set. The external validation results shown that the model is well generalized in predicting the specific abnormalities e.g. cavity, opacity, pleural effusion which are consistent with active TB. The lack of labelled Tuberculosis dataset affected the performance of metrices in external validation. The available literature in deep learning community was either binary classification of Tuberculosis, or multilabel classification of thoracic abnormalities in image wise. There is no published article in classification of active pulmonary tuberculosis manifestations in lung zone wise.

The grad-CAM analysis shows that the focus of grad-CAM is indeed on the manifestations of active tuberculosis affected areas. This localisation heatmap is remarkable for its meticulousness and can be used in clinical settings to aid the clinicians in primary health care where service from expert radiologist is often lacking or nil.

## Materials and methodology

### Study design and goal

This was a retrospective study with classification model creation using transfer learning technique to detect the various manifestations in active tuberculosis from CXR. This was followed by generation of Grad-CAM heatmap and comparison with radiologist annotated ground truth.

### Dataset sources and curation for model development

We retrospectively collected chest X-ray images and, patient demography from the National TB Elimination Programme referral register in a specific DMC from 2017 to 2020. Patients were confirmed as active TB either by physician or microbiological examinations. Posteroanterior (PA) chest X-ray was downloaded from the Picture Archival and Communication System (PACS) which use Health Level Seven International (HL7) integration system between PACS and Health Information System. Unique patient identifier was used to extract the images from PACS where the date of CXR taken was close to the date of sputum examination result date. If multiple CXR were available within the lab result date, we collected all available CXR and sequentially numbered based on the date to identify the prognosis/progress of TB. All CXR used in the study were de-identified using system generated study identifier and any overlay information in CXR were removed to protect the privacy of the patients.

There were no missing data on patient demographics and CXR and images were in Tag Image File Format with 24 Bits per pixel. Digital Radiography modality was used with resolution of 1530 × 1896. All CXR were taken by Portable Samsung Retrofit DR System. We collected 1350 CXR from 858 patients confirmed with active TB. We excluded 38 images of subjects under 15 years of age from training the model as per WHO 2021 guidelines for CAD usage in screening and triaging^4^. There were 687 repeated CXR from 212 patients with an average of 3.2 images per patients. The final dataset comprised of 1312 CXR from 837 patients. Mean age of the TB patients was 45.8 years [14.6 years standard deviation (SD)] with an age range of 15–82 years and 20.2% being female.

### Dataset sources and curation for external validation

Publicly available Health Insurance Portability and Accountability (HIPPA) compliant datasets maintained by the National Library of Medicine, Maryland, USA were used as external test set -Department of Health and Human Services, Montgomery County, Maryland, USA (MC) collection^33^. The MC collections are in in Portable Network Graphics (PNG) format with chest X-ray of 58 TB patients. We curated 36 abnormal CXR for external test set validation after excluding pediatric, pneumonia and old/inactive TB images. Mean age of MC collection was 47.5 (20.6 SD) and 33.3% being female.

### Ground truth

All PA TB CXR were read by single radiologist (HG) for various active TB manifestations and reported in a specified format approved by the study team. Peer validation of 10% CXR was done by the radiologist (SA) and pulmonologist (MR). The inter-observer agreement between reader and two peer assessors (SA and MR) was almost perfect and moderate (k = 0.83, [95% CI: 0.72–0.93] and k = 0.80, [95% CI: 0.71–0.94] respectively)^34^.

In the present study we excluded abnormalities that occur less than 10% in data points from training, validation and testing dataset. The final dataset contains X-rays with 44 labels in total—12 abnormalities in image wise,30 abnormalities in bilateral upper, middle and lower zones and 14 abnormalities reported as left and right lung findings Ground truth of the MC collection was obtained from the dataset source.

### Data partitions and augmentation

Dataset was split into training (80%) and test (20%) sets. We split the training set into training (64%) and validation (16%). Repeated images of the same patients were included only in training set to avoid data leakage in validation set and holdout test set. We performed offline augmentation on training set alone with the following parameters (1) random rotation with probability of 1 and rotation range from ± 7 degrees excluding zero (2) Random brightness with probability of 1 by adjusting the color value “v” by NumPy random uniform function from the “hsv” image format.

### Active TB manifestation classification model

**Figure 6:**
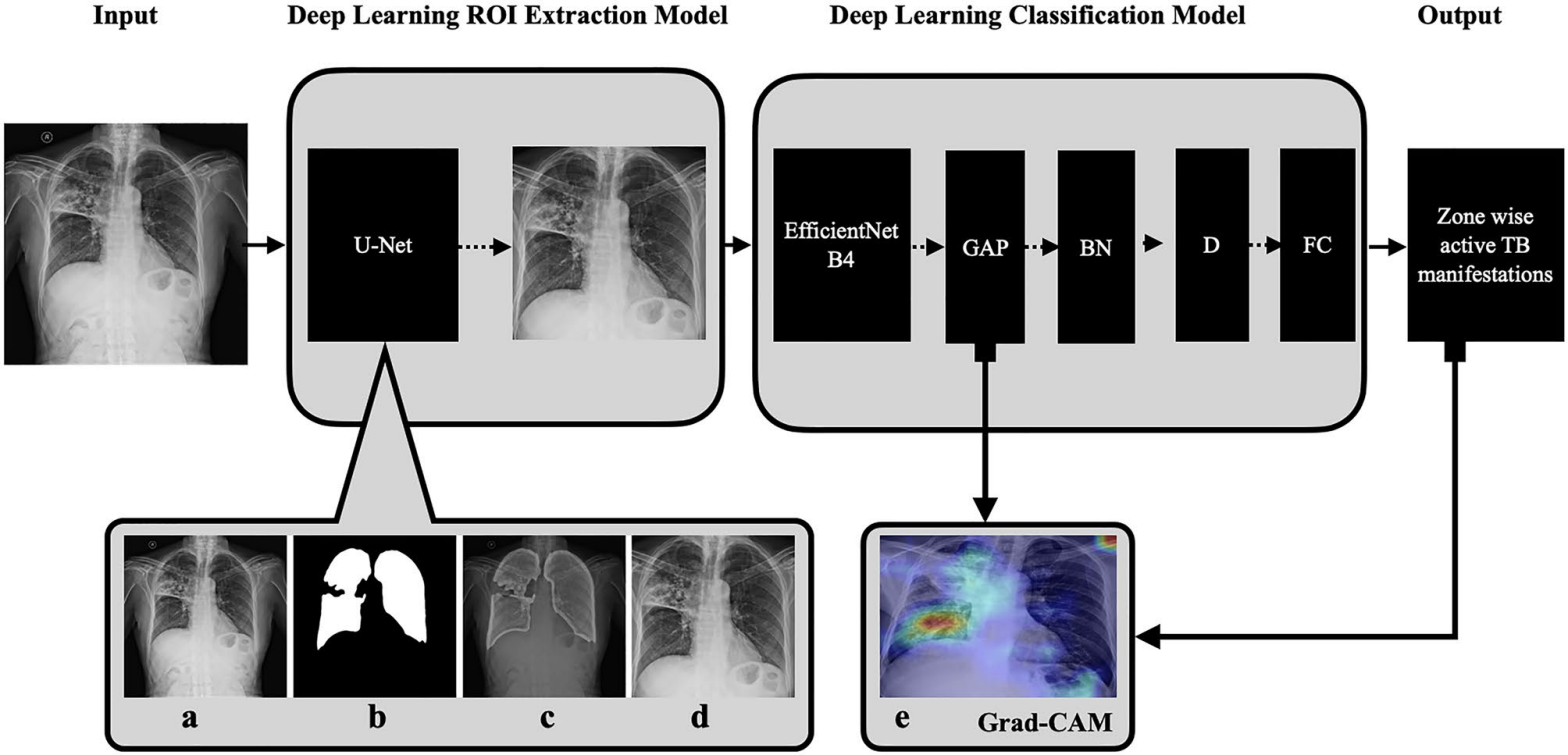
Basic outline of the network architecture. Input—image of chest radiograph. *ROI* region of interest. In the ROI extraction model: (**a**) original chest X-ray, (**b**) mask of lung generated by U-Net, (**c**) mask overlay to original chest X-ray, and (**d**) final segmented image of the lungs based on mask in 380 × 380 size. (**e**) Grad-CAM visualisation. Pre-trained model—EfficientNetB4 trained on ImageNet without the final classifier layer. *GAP* global average pooling, *BN* batch normalisation layer, *D* dropout layer, *FC* fully connected classifier with 44 outputs. Due to complexity we omitted activation layer and classifier activation functions. The outputs are parenchymal: opacity, fibrosis, collapse, cavity, calcification (lung zone wise). Pleural: thickening, pneumothorax, effusion and overall: tracheal shift, volume loss, mediastinal shift, emphysema (lung wise).

In this work, we employed U-Net for segmentation of the region of interest (ROI) and EfficientNetB4 for classification of active TB manifestations. The Deep Convolutional Neural Networks (DCNN) model was developed/fine-tuned under TensorFlow framework (version 2.7.0, Google Brain Team, CA, USA https://tensorflow.org), Keras (version 2.7.0, https://keras.io) with Python (version 3.7, Python Software Foundation, DE, USA https://python.org) as the programming language (Fig. 6). The desktop computer was equipped with Intel i9-9820X CPU @3.30 GHz, 64G RAM, and dual NVIDIA GeForce RTX 2080Ti @11G GPU.

For the Region of Interest (lung area) segmentation task, we used U-Net, a deep convolutional neural network architecture that has shown remarkable accuracy in medical image segmentation tasks. Segmented lung area was padded with 50 pixels to retain the lung border and extracted into 380 × 380 size and saved in Joint Photographic Experts Group (JPEG) format. The saved images were then partitioned, augmented and fed into classification model for training, validation and testing.

EfficientNetB4^35^ pre-trained networks on ImageNet were used in this work as base models. The input image size of EfficientNetB4 was set to 380 × 380. We used weights from ImageNet (transfer learning) to initialize the network. The classifier in base model is replaced with the following; (1) a Global Average Pooling layer (GAP), (2) a Batch Normalization layer (BN), (3) a Dropout layer (D), and (4) a classifier layer with 12/44 output classes and Sigmoid activation.

### Model training

#### Training EfficientNetB4 model with 12 class

Progressive training (Supplementary Fig. S1) was performed to the EfficientNetB4 model with 12 labels. Initially all layers except Batch Normalization layers in the EfficientNetB4 model were set non-trainable as the mean and variance of the training dataset differed from ImageNet ^36^ and initialized the network with ImageNet weights. The training was performed using Sigmoid Focal Cross Entropy as losses function and Adam as optimizer with following parameters β1 = 0.9, β2 = 0.999, ϵ = 1 × 10^–7^ and learning rate 1 × 10^–6^.

The network was trained for 50 epochs (training session 1) with mini-batches of 4 samples and Early Stopping with patience set to 3 aided by validation loss. The mini-batches were shuffled on each epoch to randomize the training method and decrease overfitting and saved the model weights in hdf5 format.

In training session 2, we re-initialized the network with saved model weights from training session one, then we unfroze top 31 layers and retrained the network for 100 epochs with learning rate reduction of 1 × 10^–1^, keeping β1, β2, ϵ, and mini-batch parameters unchanged and saved trained model weights in hdf5 format.

In training session 3, we initialized EfficientNetB4 with training session two weights and fine-tuned top 150 layers of the network with learning rate 1 × 10^–8^ and keeping β1, β2, ϵ, and mini-batch parameters unchanged for 150 epochs and saved trained model weights in hdf5 format. In the last training session, network was initialized with weights from training session three and fine-tuned top 242 layers for 200 epochs with learning rate 1 × 10^–9^ and other hyperparameters were unaltered.

#### Training EfficientNetB4 model with 44 class

The final saved weights from training session 4 were used to initialize EfficientNetB4 network and change the classifier head with 44 labels. The network was fed with dataset with 44 class label and repeated the training strategy adopted in training EfficientNetB4 model with 12 class.

### Evaluation metrics

Testing of the model was done using intramural holdout test set and extramural test set (MC collection). The following metrics were used to evaluate the classification of the model using the test sets, (1) Sensitivity, (2) Specificity, (3) Area Under the Receiver Operating Characteristic (AUC), (4) Accuracy, (5) Precision and (6) F1-Score. In MC collection, classes with zero data points were excluded from Sensitivity, AUC, Precision and F1-Score analysis. Confidence Interval (CI) for AUC were calculated using Hanley & McNeil test^37^, and CI for sensitivity and specificity were obtained using the Wilson Score^38^ method. Statistical analysis was done using Python 3.7 statistical library, and a P-value of 0.05 was considered statistically significant.

### Visualisation

We generated Grad-CAM for the trained network to understand the network and to gain the visual detection of abnormalities from CXR. This technique was used to get the discriminative image regions to weigh more for classification of the abnormalities. Grad-CAM uses deeper higher abstract feature maps to generate the heatmap, which typically produce better localisation, but there is a trade off on the quality of the images due to the pooling layers leading to coarse localisation of abnormalities. In this work we generated grad-CAM for the EfficientNetB4 multi-label network trained with 44 class. Generated grad-CAM were analysed and interpreted by the radiologist (HG).

### Strengths and limitations

This study has several strengths. The use of EfficientNetB4 architecture and the CXR interpretations and findings were peer validated by pulmonologist and radiologist and obtained inter-reader agreement. In this work, active tuberculosis manifestations were classified into lung zones and lung wise. We applied domain specific data augmentations after a detailed literature review and expert opinion from radiologist. We employed cross population data set (MC collection) to evaluate the model performance.

One of the primary limitations of the study was limited number of data points in certain classes, and we excluded data points which had prevalence less than 10%. All CXR were read by only single radiologists opposed to multiple radiologists or pulmonologists. Though a sample of findings was peer validated there may have been some degree of misclassification of the visual signals in the chest X-ray.

## Conclusions

Digital technologies, especially Artificial Intelligence based solutions can accelerate the screening, triaging, and diagnosis of Tuberculosis in automation of radiology. Deep learning algorithm can aid in interpretation of chest X-ray findings such as parenchymal and pleural abnormalities. In this work we focused on the classification of active pulmonary tuberculosis manifestations both image wise and lung zone wise. Our study demonstrates that deep learning network can classify active tuberculosis manifestations with remarkable accuracy in image wise and lung zone wise. Classification of abnormalities along with Grad-CAM visualization would be helpful in improving the precision and accuracy of the detection of tuberculosis manifestations and expedite the screening and triaging process in resource constrained settings where the service of trained technologists or expert radiologists is lacking or overburdened. As of our best knowledge this is the first work to classify thirty abnormalities categorized into upper, mid and lower zones of left and right lung, and fourteen abnormalities in left and right lung wise. Further research in the classification of various abnormalities of active tuberculosis is necessary and there is a scarcity of literature in this domain.

### Ethics approval and consent

Ethical approval for this study was obtained from the Institutional Ethics committee for Observational studies of Jawaharlal Institute of Postgraduate Medical Education and Research (JIPMER), Puducherry, India (JIPMER ethics committee number JIP/IEC/2019/533). Waiver of written informed consent was approved by JIPMER institutional ethics committee as all data sources used (patient demography, laboratory records, and chest X-ray) were previously available, and no patients needed to be contacted. Additionally, all data were collected anonymously and de-identified using study identifier before reading by the radiologist, model development, validation, and testing. All methods were carried out in accordance with Indian Council of Medical Research (ICMR) and International Committee on Harmonization of Good Clinical Practice (ICH-GCP) guidelines and regulations.

## Supplementary Information

Supplementary Information.

## Data Availability

The raw patient image data that support the findings of this work are taken from an ongoing study and due to specific institutional requirements governing privacy protection, the dataset used in this work are not available in public. The external test dataset used in this study, Montgomery is available at https://data.lhncbc.nlm.nih.gov/public/Tuberculosis-Chest-X-ray-Datasets/Montgomery-County-CXR-Set/MontgomerySet/index.html.
