# Supplementary material for: Deep learning classification of active tuberculosis lung zones wise manifestations using chest X-rays: a multi label approach
Source: Sci Rep. 2023 Jan 17;13:887. doi: 10.1038/s41598-023-28079-0 (PMC9845381; doi:10.1038/s41598-023-28079-0)
Supplement: Supplementary file 1 — Supplementary Information. [file 41598_2023_28079_MOESM1_ESM.pdf]

Title

Deep Learning Classification of Active Tuberculosis Lung Zones Wise Manifestations Using Chest X-Rays: A Multi Label approach.

Authors

James Devasia[1], Hridyanand Goswami[4], Subitha Lakshminarayanan[1*], Manju Rajaram[2], Subathra Adithan[3]

Affiliations

1. James Devasia (jamestd@gmail.com) and Subitha Lakshminarayanan (subitha.l@gmail.com) Department of Preventive and Social Medicine, Jawaharlal Institute of Postgraduate Medical Education & Research, Jipmer Campus Rd, Gorimedu, Priyadarshini Nagar, Puducherry, 605006.
2. Manju Rajaram (mail2manju22@gmail.com) Department of Pulmonary Medicine, Jawaharlal Institute of Postgraduate Medical Education & Research, Jipmer Campus Rd, Gorimedu, Priyadarshini Nagar, Puducherry, 605006.
3. Subathra Adithan (subathra26@gmail.com) Department of Radiodiagnosis, Jawaharlal Institute of Postgraduate Medical Education & Research, Jipmer Campus Rd, Gorimedu, Priyadarshini Nagar, Puducherry, 605006.
4. Hridayanand Goswami (hriday.goswami@gmail.com) Head, Department of Radiology, Marwari Hospitals, Sati Joymati Road, Athgaon, Guwahati, Assam. PIN 781008.

*Corresponding author

Supplementary Table S1 Diagnostic performance of 12 class on Test Set

Abnormality	Accuracy	Precision	F1 Score
Cavity	0.87	0.95	0.88
Opacity	1.00	1.00	1.00
Fibrosis	0.90	0.94	0.92
Calcification	0.95	0.81	0.75
Collapsed Lung	0.91	0.96	0.91
Pleural Effusion	0.99	0.95	0.93
Pleural Thickening	0.91	0.93	0.91
Pneumo/Hydropneumothorax	1.00	1.00	0.96
Tracheal Shift	0.90	0.92	0.86
Mediastinal Shift	0.96	0.95	0.81
Volume Loss	0.94	0.92	0.89
Emphysema Hyperinflations	0.98	1.00	0.93

Supplementary Table S2 Diagnostic performance of 44 class on Test Set

Abnormality	Accuracy	Precision	F1 Score
Cavity RUZ	0.81	0.74	0.71
Cavity LUZ	0.82	0.66	0.68
Cavity RMZ	0.84	0.56	0.55
Cavity LMZ	0.84	0.59	0.50
Cavity RLZ	0.95	0.00	-
Cavity LLZ	0.97	0.00	-
Opacity RUZ	0.80	0.91	0.87
Opacity LUZ	0.82	0.96	0.86
Opacity RMZ	0.74	0.85	0.82
Opacity LMZ	0.84	0.92	0.89
Opacity RLZ	0.74	0.82	0.73
Opacity LLZ	0.78	0.88	0.78
Fibrosis RUZ	0.86	0.86	0.83
Fibrosis LUZ	0.84	0.80	0.76
Fibrosis RMZ	0.79	0.55	0.52
Fibrosis LMZ	0.71	0.48	0.53
Fibrosis RLZ	0.88	0.33	0.27
Fibrosis LLZ	0.88	0.67	0.34
Calcification RUZ	0.95	0.71	0.68
Calcification LUZ	0.94	0.53	0.53
Calcification RMZ	0.96	0.79	0.75
Calcification LMZ	0.94	0.60	0.53
Calcification RLZ	0.97	1.00	0.31
Calcification LLZ	0.97	0.00	-
Collapsed Lung RUZ	0.84	0.77	0.74
Collapsed Lung LUZ	0.89	0.81	0.77
Collapsed Lung RMZ	0.86	0.43	0.39
Collapsed Lung LMZ	0.90	0.63	0.66
Collapsed Lung RLZ	0.92	0.53	0.49
Collapsed Lung LLZ	0.91	0.69	0.61
Pleural Effusion R	0.95	0.46	0.63
Pleural Effusion L	0.98	0.73	0.81
Pleural Thickening R	0.84	0.77	0.73
Pleural Thickening L	0.89	0.77	0.81
Pneumo/Hydropneumothorax R	0.99	0.86	0.80
Pneumo/Hydropneumothorax L	0.98	0.63	0.72
Tracheal Shift R	0.88	0.77	0.77
Tracheal Shift L	0.95	0.81	0.79
Mediastinal Shift R	0.97	0.73	0.67
Mediastinal Shift L	0.96	0.71	0.71
Volume Loss R	0.93	0.81	0.78
Volume Loss L	0.97	0.88	0.89
Emphysema R	0.96	0.76	0.82
Emphysema L	0.97	0.77	0.84

RUZ – Right Upper Zone, RMZ – Right Mid Zone, RLZ – Right Lower Zone, LUZ – Left Upper Zone, LMZ – Left Mid Zone, LLZ – Left Lower Zone, R – Right Lung, L – Left Lung. – division error (No True Positives).

Supplementary Table S3 Diagnostic performance of 44 class on External Test Set

Abnormality	Accuracy	Precision	F1 Score
Cavity RUZ	0.86	1	0.62
Cavity LUZ	0.89	0.5	0.33
Cavity RMZ	0.97	0	-
Cavity LMZ	0.92	-	-
Cavity RLZ	1	-	-
Cavity LLZ	1	-	-
Opacity RUZ	0.69	0.53	0.65
Opacity LUZ	0.86	0.83	0.67
Opacity RMZ	0.72	0.44	0.61
Opacity LMZ	0.94	0.75	0.86
Opacity RLZ	0.64	0.28	0.44
Opacity LLZ	0.94	0.33	0.5
Fibrosis RUZ	0.81	0.5	0.53
Fibrosis LUZ	0.81	0.4	0.53
Fibrosis RMZ	0.89	0.33	0.5
Fibrosis LMZ	0.75	0.18	0.31
Fibrosis RLZ	0.94	0	-
Fibrosis LLZ	1	-	-
Calcification RUZ	1	-	-
Calcification LUZ	0.94	-	-
Calcification RMZ	1	-	-
Calcification LMZ	0.97	-	-
Calcification RLZ	0.97	-	-
Calcification LLZ	1	-	-
Collapsed Lung RUZ	0.92	0	-
Collapsed Lung LUZ	0.97	0.5	0.67
Collapsed Lung RMZ	0.94	0.33	0.5
Collapsed Lung LMZ	1	-	-
Collapsed Lung RLZ	0.97	0	-
Collapsed Lung LLZ	1	-	-
Pleural Effusion R	1	1	1
Pleural Effusion L	0.97	1	0.8
Pleural Thickening R	0.89	0.25	0.33
Pleural Thickening L	0.97	0.67	0.8
Pneumo/Hydropneumothorax R	1	-	-
Pneumo/Hydropneumothorax L	1	-	-
Tracheal Shift R	0.94	0.75	0.75
Tracheal Shift L	1	-	-
Mediastinal Shift R	1	1	1
Mediastinal Shift L	1	-	-
Volume Loss R	0.97	-	-
Volume Loss L	0.97	0.5	0.67
Emphysema R	0.94	0	-
Emphysema L	0.89	0	-

RUZ – Right Upper Zone, RMZ – Right Mid Zone, RLZ – Right Lower Zone, LUZ – Left Upper Zone, LMZ – Left Mid Zone, LLZ – Left Lower Zone, R – Right Lung, L – Left Lung. – division error (No True Positives).

Supplementary Figure S1 Block diagram of Progressive training of EfficientNetB4 model

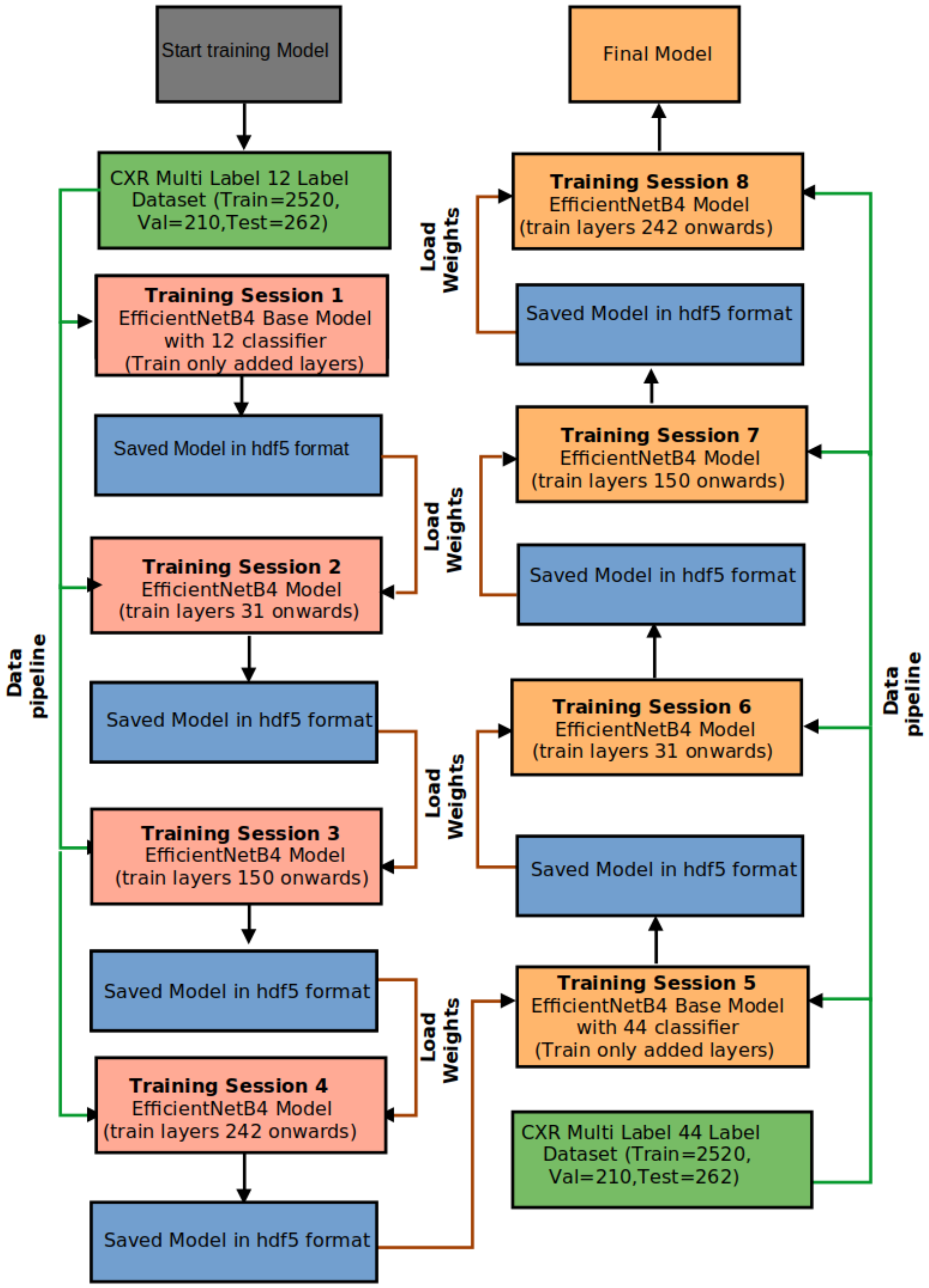

Supplementary Figure S2 GradCAM images of intramural test set on EfficientNetB4 model

Input CXR	Ground Truth	grad-CAM	Predicted Class with Score
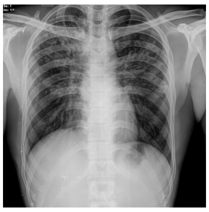	Cavity RUZ,LUZ<br>Opacity RUZ, LUZ, RMZ, LMZ<br>Fibrosis RUZ, LUZ, RMZ, LMZ	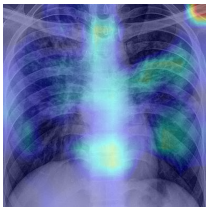	Cavity RUZ (0.87), LUZ (0.40), LMZ (0.53)<br>Opacity RUZ (0.99), LUZ (0.89), RMZ (0.87), LMZ (0.93), LLZ (0.94)<br>Fibrosis RUZ (0.95), LUZ (0.33), RMZ (0.37), LMZ (0.21)
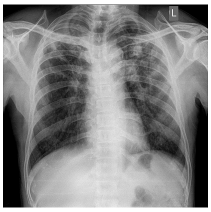	Cavity LMZ<br>Opacity RUZ, LUZ, LMZ<br>Collapsed Lung RUZ<br>Pleural Thickening R<br>Tracheal Shift R<br>Volume Loss R	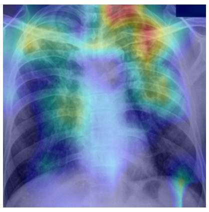	Cavity RUZ (0.93), LUZ (0.94), LMZ (0.0)<br>Opacity RUZ (0.86), LUZ (0.99), RMZ (0.68),LMZ (0.96)<br>Collapsed Lung RUZ (0.93), LUZ (0.72)<br>Pleural Thickening R (0.66)<br>Tracheal Shift R (0.93)<br>Volume Loss R (0.99)
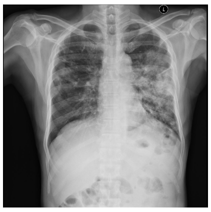	Opacity LUZ, RMZ, LMZ, LLZ<br>Fibrosis RMZ	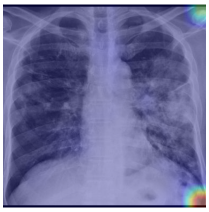	Opacity LUZ (0.50), RMZ (0.63), LMZ (0.99), LLZ (0.50)<br>Fibrosis RMZ (0.29), LMZ (0.52)
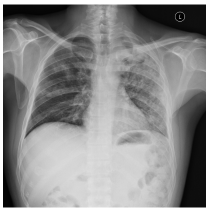	Cavity RUZ<br>Opacity RUZ, LUZ, RMZ, LMZ, LLZ<br>Collapsed Lung LUZ<br>Tracheal Shift R	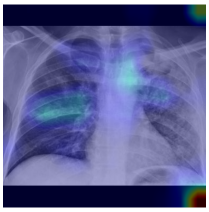	Cavity LUZ (0.57), RUZ (0.42)<br>Opacity RUZ (0.43), LUZ (0.84), RMZ (0.33), LMZ (0.99), LLZ (0.92), RLZ (0.58)<br>Collapsed Lung LUZ (0.98), LMZ (0.81)<br>Tracheal Shift R (0.92)
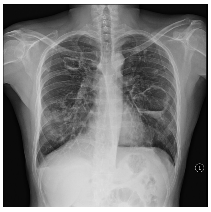	Cavity RUZ, LMZ<br>Opacity RUZ, LUZ, RMZ, LMZ, RLZ<br>Fibrosis RUZ, LUZ, RMZ	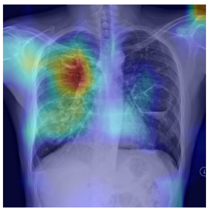	Cavity RUZ (0.57), LMZ (0.1), LUZ (0.82)<br>Opacity RUZ (0.63), LUZ (0.21), RMZ (0.25), LMZ (0.79), RLZ (0.78), LLZ (0.79)<br>Fibrosis RUZ (0.59), LUZ (0.82), RMZ (0.59), LMZ (0.75)
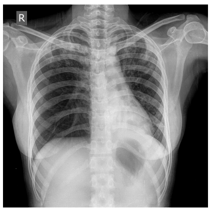	Opacity RUZ, LUZ, LMZ, LLZ<br>Fibrosis RUZ, LUZ<br>Collapsed Lung LUZ, LLZ<br>Pleural Thickening L<br>Mediastinal Shift L<br>Volume Loss L	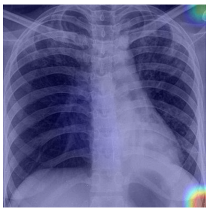	Opacity RUZ (0.59), LUZ (0.99), RMZ (0.52), LMZ (0.97), LLZ (0.83)<br>Fibrosis RUZ (0.78), LUZ (0.97), LMZ (0.83)<br>Collapsed Lung LUZ (0.95), LLZ (0.76)<br>Pleural Thickening L (0.98)<br>Mediastinal Shift L (0.83), R (0.91)<br>Volume Loss L (0.77)
